# A study on the current situation and influencing factors of childcare service demand for parents of infants aged 0–3 years in Ningbo city of China

**DOI:** 10.3389/fpubh.2026.1752192

**Published:** 2026-06-30

**Authors:** Ning Sun, Hongyu Li, Chenchen Zhu, Kaiying Zhong, Shuang Yang, Qianru Li

**Affiliations:** School of Nursing, Ningbo College of Health Sciences, Ningbo, China

**Keywords:** childbearing age, childcare services, children aged 0–3, demand, influencing factors, people

## Abstract

**Objectives:**

Amid China's population structure adjustment, shortage of high-quality, accessible childcare services for 0–3 years old constrains fertility willingness and family well being. Existing studies lack targeted analysis on demand structure and influencing mechanisms in urban areas. Therefore, this study investigated the characteristics of childcare service demand and key socioeconomic determinants among Ningbo's childbearing population. These results provide empirical support for optimizing regional childcare policies.

**Methods:**

A self-designed questionnaire was administered online in Ningbo, China from July to September, 2024. Information on personal and family characteristics, as well as demand for childcare services, was obtained. Descriptive statistics and multiple linear regression were used to analyze the determinants of childcare services.

**Results:**

Of the 320 questionnaires distributed, 300 had valid responses (response rate of 93.8%). The seven dimensions of childcare service demand were ranked from highest to lowest: convenient conditions (9.39 ± 4.39 points), early education (8.86 ± 4.60), environmental facilities (8.60 ± 4.67), teaching staff quality (8.57 ± 4.23), childcare center management (8.40 ± 4.64), home-care interaction (7.73 ± 3.72), and childcare services (5.73 ± 3.04 points). Analysis revealed three variables in the regression model: parents' age, occupation, and educational background (*p* < 0.001).

**Conclusion:**

Demand for childcare services was influenced by multiple factors. Innovating cooperation models that optimize policy support and strengthen new supportive measures can help provide convenient childcare services. Furthermore, enhancing government-industry supervision and positive community publicity, improving early education policies, and developing service models can increase the demand and level of childcare services in China.

## Background

On July 20, 2021, the Central Committee of the Communist Party of China and State Council issued the Decision on Optimizing Birth Policies to Promote Long-term Balanced Population Development, officially ushering China into the “three-child policy” era (1). However, the sustained low birth rate and limited effectiveness of fertility-support policies highlight the urgency of improving infant and toddler childcare services—defined as “preschool care and scientific parenting guidance for children under 3 years old and their parents” (2). Long-term population restriction policy, fertility willingness, work-family balance, and intergenerational expectations are key factors of livelihood development that impact family fertility (3). Although China's childcare service demand has recently diversified, a prominent supply-demand mismatch persists: a large gap exists between families' strong demand for high-quality childcare and actual enrollment rates, with many unmet needs (3). Thus, exploring the specific demand structure of childcare services among the childbearing population and identifying its influencing factors are essential for optimizing policies, bridging gaps, and supporting national fertility strategies.

Existing studies have explored parental demand for childcare services from multiple dimensions. Regarding demand priorities, service convenience (e.g., proximity to home), quality (e.g., teacher professionalism, facility environment), early education content, and management standards were identified as key concerns (3–5). Peng (3) categorized childcare demands into early education, management, interaction, and convenience (3). Furthermore, Li et al. (4) emphasized parents' requirements for spacious activity spaces and professional guidance (4) while Fei and Jiao (5) highlighted healthcare and teaching staff quality as top priorities (5). Regarding service format and cost, parents prefer public or community-based childcare centers, with monthly fees ranging from 1,000–2,999 yuan, and value additional support, such as scientific parenting lectures (6–8).

Regarding influencing factors, parents' socioeconomic characteristics (e.g., educational background, occupation, and income) and family attributes (e.g., household income, work status) are well-documented determinants (9–11). Higher educational attainment and full-time employment increase parents' preference for formal childcare services (11, 12) as high-income families are more willing to invest in paid childcare (13). Regional disparities (e.g., urban-rural, city scale) also shape demand intensity (14). Additionally, parental age, gender, and ethnic background affect service preferences, such as younger mothers with higher education demanding better service quality (15), and immigrant families favoring home-based care (16).

However, critical research gaps remain: (1) Most studies have focused on general demand characteristics and lack systematic analysis of demand structure in emerging urban areas (e.g., prefecture-level cities, such as Ningbo). (2) Furthermore, existing research on the influencing factors is fragmented, with inconsistent conclusions on the relative importance of key variables (e.g., age vs. occupation vs. education) in specific regional contexts. (3) Few studies have explicitly linked demand characteristics to policy optimization and fail to provide targeted empirical support for local childcare service system improvement. These gaps limit the effectiveness of policies designed to address supply-demand mismatches.

Therefore, this study addressed two core questions: What is the priority ranking of demand dimensions (admission convenience, early education, environmental facilities, etc.) among the childbearing population in Ningbo, China for 0–3-year-old childcare services? and Which socioeconomic factors (parental age, occupation, educational background, etc.) significantly influence the intensity of childcare service demand, and what are their respective effects?

This study aimed to systematically investigate the demand structure and key factors that influenced 0–3-year-old childcare services among Ningbo's childbearing population. These results can provide targeted empirical evidence for optimizing regional childcare service policies and bridging supply-demand gaps.

Based on existing literature and research gaps, we proposed two integrated hypotheses:

*H1:* Convenient conditions and early education would be the main demand dimensions for 0–3 years old childcare services for Ningbo's childbearing population.

*H2:* Parental educational background, occupation, age, and education would be positively correlated with childcare service demand intensity.

This study has both theoretical and practical contributions. Theoretically, it enriches literature on childcare service demand in urban areas, clarifies the relative importance of the key influencing factors, and provides a framework for regional-specific demand analysis. Practically, these findings can guide local governments to optimize childcare service supply (e.g., prioritizing convenient location layout, improving early education content) and formulate targeted policies (e.g., subsidizing low-income families, strengthening teacher training). Thus, access to and quality of childcare services can be enhanced, supporting family fertility decisions, and promoting long-term population balanced development.

## Methods

### Study design

A correlational cross-sectional design was adopted and questionnaires were used for data collection.

### Participants

Convenience sampling was used to obtain a list of participants from Ningbo City, China between July and September 2024. The birth rate in Ningbo has been declining year-by-year. Ningbo officially launched the construction of a child-friendly city in November 2021. On April 14, 2023, the National Development and Reform Commission and the Office of the National Working Committee on Children and Women under the State Council jointly issued a document, formally including Ningbo in the second batch of national child-friendly city construction list. This study was a regional exploratory analysis. **T**he questionnaire was administered to 320 participants. Inclusion criteria were those: (1) aged 15–49 years, (2) capable of independently understanding and completing the questionnaire in Chinese, and (3) proficient in using digital devices, such as smartphones, tablets, or computers for online surveys. Exclusion criteria were individuals: (1) aged >15 or >49 years, (2) who had communication or cognitive impairments that prevented them completing the questionnaire, and (3) were unable to use digital devices to participate in the online survey.

### Sample size calculation

This study examined the correlation between individual factors and childcare service demands. Multi-factor analysis methods were applied. Based on relevant research, 18 variables could be entered into the model. The required sample size was estimated to be at least 10–15 times the number of variables entered. Thus, 270 participants were required. Based on a follow-up loss rate of 10%, the required sample size was approximately 297 people.

### Data collection

Two investigators underwent unified training for data collection. Eligible participants were invited to complete an online questionnaire through a secure electronic data collection platform. The study's objectives and methods were explained to those who met the inclusion criteria. Furthermore, informed consent was obtained online. Each completed questionnaire was assigned a code to ensure anonymity.

### Instruments

Data were obtained for three domains: socio-demographic characteristics, childcare model demand, and childcare service demand.

Questionnaire items were obtained *via* a literature review and expert interviews. An expert panel (five experts) was established to assess content validity. The five experts were selected based on the following criteria: (a) had ≥5 years of experience in caring for child, (b) worked in childcare service centers, and (c) had a title of deputy senior or higher. In addition, all experts answered a final question regarding the relevance of the entire questionnaire.

Demographic details included infants' age, parents' age, their educational background, whether the parents were only children, their occupation, monthly household income, whether the grandparents cared for the infants, grandparents' health status, and family composition.

The childcare model demand questionnaire was independently developed by the research team to assess the demand levels for childcare models among different age groups: 0–11, 12–23, and 24–36 months. Items were rated on a 5-point Likert scale ranging from 5 (very much needed) to 1 (very much not needed). Higher scores reflected stronger demand for the childcare model. In this study, the questionnaire demonstrated Cronbach's alpha reliability of 0.96 and content validity of 0.94. A confirmatory factor analysis was conducted and yielded extremely significant results: chi-square (χ^2^) = 1,367.526, chi-square/degrees of freedom (χ^2^/df) = 2.056, comparative fit index (CFI) = 0.967, root mean square error of approximation (RMSEA) = 0.045, and *p* < 0.05.

The Infant and Toddler Care Service Needs Questionnaire, independently developed by our research team, comprised seven primary (six items each for environmental facilities, childcare center management, childcare services, early education, teaching staff, convenient conditions, and home-care interaction) and 39 secondary indicators. Responses were rated on 5-point Likert scale ranging from 5 (very important) to 1 (very unimportant). Higher scores reflected greater family demand. In this study, Cronbach's alpha reliability was 0.99 and content validity was 0.95. A confirmatory factor analysis was conducted and yielded extremely significant results: χ^2^ = 1,236.627, χ^2^/df = 2.126, CFI = 0.978, RMSEA = 0.046, and *p* < 0.05.

### Ethical considerations

This study was approved by the Institutional Review Board of Ningbo College of Health Sciences. All participants provided informed consent before participating online. Two researchers were responsible for informing the participants of the study's the purpose and data collection. Individuals were informed that participation was voluntary and withdrawal would have no negative consequences.

### Data analysis

This study utilized Microsoft Excel for database construction and SPSS version 27.0 for statistical analysis, with significance levels set at *P* < 0.05. Descriptive statistics were employed to analyze demographic characteristics and related variables and presented as means ± standard deviation for quantitative data and frequency (n) with percentage composition for categorical data. A multiple linear regression was used to investigate the relationship between participants' demographic characteristics and childcare service demand.

## Results

### Participants' demographics

Of the 320 questionnaires distributed, 300 had valid responses (a response rate of 93.8%). Infants' and parents ages ranged from 1–36 months (average 13.56 ± 3.87 months) to 24–49 years (average 30.12 ± 2.97 years), respectively. Most parents held bachelor's degrees (46.0%), and 60% were single children. Regarding occupation, 29.0% were professional technicians. Furthermore, 66.0% of families had a monthly income of >5,000 yuan. In addition, 71.0% of the infants lived with their grandparents, 71.3% of whom were in average health conditions, and 67.3% of families were nuclear households.

### Analysis of current demand for childcare models

In the childcare service demand survey, families with infants aged 0–11, 12–23, and 24–36 months all expressed preference for institutional childcare services, and scored 2.80 ± 1.35, 2.55 ± 1.20, and 2.43 ± 1.20 respectively. Regarding childcare duration, families favored full-day care services across all age groups, with scores of 3.11 ± 1.38, 2.90 ± 1.29, and 2.82 ± 1.30 respectively. Table 1 presents the detailed scores.

**Table 1:** Scores of the childcare model demand levels (*n* = 300).

Project	0–11 months	12–23 months	24–36 months
Childcare environment			
Institutional childcare	2.80 ± 1.35	2.55 ± 1.20	2.43 ± 1.20
Home-based childcare	2.33 ± 1.30	2.43 ± 1.19	2.24 ± 1.13
Childcare hours			
Daycare	2.62 ± 1.32	2.41 ± 1.19	2.21 ± 1.09
Half-day childcare	2.76 ± 1.30	2.58 ± 1.19	2.43 ± 1.15
Full-day childcare	3.11 ± 1.38	2.90 ± 1.29	2.82 ± 1.30
Temporary childcare	2.32 ± 1.26	2.27 ± 1.19	2.16 ± 1.14

### Analysis of the current situation of childcare service demand

Childcare service demand ranged from 39 to 195 points, with an average score of 57.28 ± 27.46. The seven dimensions were scored as follows: environmental facilities (8.60 ± 4.67), childcare center management (8.40 ± 4.64), childcare services (5.73 ± 3.04), early education (8.86 ± 4.60), teaching staff (8.57 ± 4.23), convenient conditions (9.39 ± 4.39), and home-care interaction (7.73 ± 3.72). Table 2 presents further details.

**Table 2:** Scores of the various dimensions of childcare service demand (*n* = 300).

Project	Score range	Mean and standard deviation
*Overall demand score*	39–195	57.28 ± 27.46
*Environmental facilities*	6–30	8.60 ± 4.67
1. Regular disinfection and cleaning of the organization, environmental safety, and hygiene	1–5	1.43 ± 0.82
2. Institution has low noise and no pollution around it; the indoor environment is comfortable and warm with good air circulation	1–5	1.45 ± 0.84
3. Facilities within the organization are sturdy and safe, with no tilting, falling off, or cracks	1–5	1.41 ± 0.79
4. Institution is equipped with sufficient books, toys, and activity equipment	1–5	1.45 ± 0.82
5. Interior features distinct activity zones for studying, recreation, and sleeping, while the exterior offers a spacious, independent area for free play	1–5	1.45 ± 0.80
6. Nursery institution is equipped with a full coverage monitoring system	1–5	1.40 ± 0.78
*Childcare center management*	6–30	8.40 ± 4.64
1. Have complete business qualifications	1–5	1.40 ± 0.78
2. Regularly disclose the organization's fee standards, nutritional menus, and hygiene safety measures to ensure parental oversight	1–5	1.40 ± 0.80
3. Health record management for practitioners	1–5	1.39 ± 0.77
4. Manage information on infants and young children entrusted to the institution, collect and update it in a timely manner, and regularly report it to the filing department	1–5	1.41 ± 0.80
5. Childcare institutions can effectively handle emergencies, such as infectious disease isolation and violent incidents	1–5	1.38 ± 0.76
6. Well-established pick-up and drop-off system is in place, with infants being picked up by their guardians or authorized adults	1–5	1.40 ± 0.81
*Childcare services*	4–20	5.73 ± 3.04
1. Make scientific diet and dietary plan to ensure balanced nutrition for infants and young children	1–5	1.40 ± 0.77
2. Provide play activities for infants and young children who do not nap, with teachers supervising them	1–5	1.50 ± 0.84
3. Feeding environment for infants and young children is clean and hygienic, and they are assisted in eating	1–5	1.40 ± 0.76
4. Scientifically arrange the life of infants and young children, such as drinking water, feeding, toilet, sleep, etc.	1–5	1.43 ± 0.81
*Early education*	6–30	8.86 ± 4.60
1. Guide infants and toddlers to actively interact with peers or adults	1–5	1.45 ± 0.78
2. Offer early education courses in painting, music, dance, etc.	1–5	1.60 ± 0.90
3. Develop courses or activities to cultivate infants' language comprehension and expression skills	1–5	1.47 ± 0.78
4. Cultivate infants' habits of hygiene, cleanliness, and politeness	1–5	1.45 ± 0.81
5. Cultivate infants' self-care ability	1–5	1.44 ± 0.80
6. Develop infants and young children's gross and fine motor skills	1–5	1.45 ± 0.81
*Teaching staff*	6–30	8.57 ± 4.23
1. Teachers have relevant qualification certificates	1–5	1.39 ± 0.76
2. Have a professional in infant care	1–5	1.51 ± 0.82
3. Teaching staff is stable and resignation rate is low	1–5	1.47 ± 0.81
4. Teachers are careful, patient, and responsible in dealing with infants and young children	1–5	1.37 ± 0.73
5. Experienced teachers	1–5	1.44 ± 0.75
6. Teachers constantly monitor infants' health, emotional states, and behavioral changes, and promptly and appropriately address them	1–5	1.39 ± 0.73
*Convenient conditions*	6–30	9.39 ± 4.39
1. Childcare is close to home or work	1–5	1.43 ± 0.75
2. Provide school bus service	1–5	1.83 ± 0.99
3. Can provide temporary or flexible childcare	1–5	1.50 ± 0.77
4. Convenient transportation near childcare institutions	1–5	1.50 ± 0.78
5. Provide convenient conditions, such as parking space for parents to pick up and drop off	1–5	1.49 ± 0.80
6. You can go to the nursery on weekends or during summer and winter vacations	1–5	1.65 ± 0.92
*Home care interaction*	5–25	7.73 ± 3.72
1. Organization guides parents to learn about infant and child care	1–5	1.50 ± 0.76
2. Institution guides parents to learn about early childhood education	1–5	1.47 ± 0.76
3. Organization keeps close contact with parents and holds regular parent meetings	1–5	1.61 ± 0.86
4. Organization provides guidance services to parents through home visits, expert lectures, parent-child activities, and other methods.	1–5	1.61 ± 0.85
5. Provide regular updates on infants' development through online interactions and face-to-face meetings	1–5	1.54 ± 0.81

### Multiple linear regression analysis of childcare service demand

Multiple linear stepwise regression analysis was used to further explore the influence of each variable on childcare service demand, with childcare service demand as the dependent variable and statistically significant variables as independent variables (α = 0.05, α = 0.1). All key linear regression assumptions (linearity, normality of residuals et al.) were systematically assessed *via* visual plots and quantitative tests. Table 3 presents the assignments of the independent variables.

**Table 3:** Assignment table of the multiple linear regressors (*n* = 300).

Variable	Number	Assignment description
Childcare service demand	Y	Original value is replaced
Infant age in months	X 1	0–11 = 1, 12–23 = 2, and 24–36 = 3
Parent age	X 2	15–25 = 1, 26–30 = 2, 31–35 = 3, and 36–49 = 4
Parental education	X 3	Junior high school or below = 1, secondary vocational school or high school = 2, college = 3, bachelor's degree = 4, master's degree or above =5
Whether parents are only children	X 4	Yes = (1,0), No = (0,1)
Parental occupation	X 5	Civil servants = (1,0,0,0,0), public institution personnel = (0,1,0,0,0), professional and technical personnel = (0,0,1,0,0), freelancers = (0,0,0,1,0), migrant workers = (0,0,0,0,1), unemployed or jobless = (0,0,0,0,0)
Monthly household income	X 6	≤ 2,000 yuan = 1, 2,001–4,000 yuan = 2, 4,001–6,000 yuan = 3, 6,001–8,000 yuan = 4, 8,001–10,000 yuan = 5, >10,000 yuan = 6
Whether infants and young children live with their grandparents	X 7	Yes = (1,0), No = (0,1)
Health status of grandparents	X 8	Good = 1, fair = 2, poor = 3
Family structure	X 9	Core family = (0,0), main family = (1,0), joint family = (0,1)

Furthermore, three significant variables were revealed: parental age, occupation, and educational level (*p* < 0.001). Analysis of variance confirmed the model's statistical significance, *F* = 25.785 (*p* < 0.001), see Table 4 for details.

**Table 4:** Multiple linear regression analysis of childcare service demand (*n* = 300).

Argument	β	SE	β^′^	*t*	*P price*
Constant	97.296	8.188		11.883	< 0.001
Age	7.027	1.569	0.252	4.478	< 0.001
Occupation	4.632	1.096	0.166	2.856	< 0.001
Record of formal schooling	4.536	1.527	0.127	2.229	< 0.001

SE, standard error.

## Discussion

### Parents' general data analysis

Parents' were aged 24–49 years, with an average of 30.12 ± 2.97 years. Among them, 138 (46.0%) held a bachelor's degree. Most were born after the 1980s, and 60% were only children. Most (29.0%) worked as professionals, such as doctors or teachers. Furthermore, 198 (66.0%) had a monthly income of >5,000 yuan. In addition, 71.0% of infants lived with their grandparents, although only 23 grandparents (7.7%) were in good health. The family structure was predominantly nuclear (67.3%). Empirical data has confirmed that post-1980s only children are the main group seeking childcare services. Despite their relatively high incomes, they still rely on intergenerational cohabitation for childcare support (17, 18).

### Situation analysis of childcare demand patterns

In the childcare service demand patterns, families with infants aged 0–11, 12–23, and 24–36 months all prioritized institutional childcare services, scoring 2.80 ± 1.35, 2.55 ± 1.20, and 2.43 ± 1.20 points, respectively. Results indicated that parents with younger infants had a stronger preference for institutional care. Furthermore, families preferred full-day childcare services. Thus, parents of younger infants favor full-day care models. These findings align with those of existing domestic and international studies that revealed families generally seek professional childcare institutions that offer full-day services for infant care. Particularly, families emphasize the need for further specialized care to promote healthy growth and development in younger infants (19–21).

### Analysis of current demand for childcare services

This study revealed that convenient conditions (9.39 ± 4.39 points), early education (8.86 ± 4.60), and environmental facilities (8.60 ± 4.67) were the three highest dimensions of childcare service demand. These findings align with those of domestic and international studies (22–24). Parents prioritize convenient conditions (school bus services scored highest: 1.83 ± 0.99, addressing work-childcare scheduling conflicts), early education (art/music/dance courses: 1.60 ± 0.90, reflecting holistic development needs), and safe environmental facilities (quiet, pollution-free, and well-ventilated spaces: 1.45 ± 0.84), highlighting safety as a core quality prerequisite. This is primarily driven by the prevalent dual-income family structure in urban China, where working parents face intense time pressure and rely on efficient childcare support for family-career balance. Additionally, since most participating parents were highly educated and valued scientific parenting, they recognized that early exposure to aesthetic and expressive courses lays a foundation for children's all-round development; furthermore, a safe and healthy environment was indispensable for infants' vulnerable physical and mental growth—consistent with their in-depth understanding of early developmental stages.

### Analysis of factors affecting childcare service demand

Results of the multiple linear regression analysis indicated three significant variables: parents' age, occupation, and educational background (*p* < 0.001). Older parents tend to have greater demands for childcare services. Parents born in the 1980s have different parenting approaches. Most new parents seek professional childcare services to support their children's healthy development. Professionals, such as doctors, lawyers, and teachers, have higher demands for comprehensive childcare services due to their elevated socioeconomic status and greater emphasis on child development. Parents with higher educational qualifications also exhibit stronger needs for childcare services, as they possess deeper understanding of infants' development patterns. They recognize that the 0–36 month period has critical developmental phases—particularly in language acquisition, imagination, and sensory perception; thus, specialized childcare services is essential for promoting infants' growth. These findings align with those of previous studies (25–27).

Notably, other theoretically relevant variables, including income, family structure, and grandparents' caregiving, were not statistically significant. Although the sample had a relatively high income level (66.0% with monthly income >5,000 yuan), income had no significant association with childcare service demand. This may be explained by the “income threshold effect”—when household income exceeds the basic cost of formal childcare services, the impact of income on demand intensity weakens (28). Furthermore, the non-significance of family structure may be due to two factors. The widespread intergenerational care support (71.0% of infants lived with grandparents) offsets potential demand differences between family structures (29). Although most infants lived with grandparents, grandparents' poor health status (only 7.7% in good health) may reduce the substitutability of informal care for formal services.

### Theoretical contributions and implications

This study has several unique contributions. First, it clarifies the demand priority structure of Chinese urban families with infants aged 0–36 months. Results highlight that “admission convenience” is the most critical factor—an insight that supplements prior research focusing on care quality and early education (22–24). This finding reflects the practical needs of working parents facing work-childcare conflicts, providing a new perspective for service design. Second, we identified age-specific demand characteristics, which can guide targeted service supply.

The Family Decision-Making Theory indicates that **t**he prioritization of admission convenience and early education reflects a “utility-maximizing” decision process. Working parents trade off-time costs (e.g., commuting for childcare) and child development benefits, choosing services that minimize opportunity costs (e.g., school bus services reducing commuting time) while maximizing developmental gains (e.g., art and music courses promoting holistic development). This extends the application of the family decision-making theory to Chinese childcare services, emphasizing the role of time cost in demand formation (30).

This study has practical implications for service design and policy-making. (1) Service accessibility should be optimized by expanding school bus coverage and simplifying admission procedures to reduce the time cost for working parents. (2) Furthermore, age-specific service design, such as enhancing specialized care for infants < 1 year old, should be strengthened and targeted early education courses should be developed. (3) Safety and professionalism in facility and staff training should be prioritized as these are core components of quality care.

### Limitations and future research

This study has some limitations. First, convenience sampling and self-reports may cause selection bias and common-method variance. Thus, future studies should adopt longitudinal, multi-source data to verify causal relationships. Second, single-city data limits the generalizability of our results. Future studies should utilize larger, diverse samples (multiple cities/rural areas) to assess the model's universality. Third, we had limited measurement of demand dimensions (e.g., incomplete early education content coverage). Thus, future research should refine these indicators and use a larger and diverse sample to verify the proposed model.

## Conclusion

China has a significant gap between the current supply and demand of childcare services. Parents prefer institutional childcare regarding type and full-day childcare regarding time. Thus, demand for childcare services places high emphasis on convenience, early education, and environmental facilities. Parents' age, education level, and occupation influence their demand for childcare services. Therefore, cooperation models should provide convenient childcare services to meet the demand for childcare services. Furthermore, policy support should be optimized and new supportive measures should be developed. Government-industry supervision and positive community publicity should be enhanced. In addition, early education policies should be improved and innovative service models should be developed.

## Data Availability

The raw data supporting the conclusions of this article will be made available by the authors, without undue reservation.
